# Group-based trajectory and predictors of anxiety and depression among Chinese breast cancer patients

**DOI:** 10.3389/fpubh.2022.1002341

**Published:** 2022-10-10

**Authors:** Wengao Li, Qiongxiao Zhang, Yining Xu, Hengwen Sun, Youlu Wen, Wenjing Xu, Yiling Tong, Samradhvi Garg, Yu Chen, Yuan Yang

**Affiliations:** ^1^Department of Psychiatry, Guangdong 999 Brain Hospital, Guangzhou, China; ^2^Department of Nursing, Guangzhou First People's Hospital, The Second Affiliated Hospital of South China University of Technology, Guangzhou, China; ^3^Department of Radiotherapy, Cancer Center, Guangdong Provincial People's Hospital (Guangdong Academy of Medical Sciences), Guangzhou, China; ^4^The Second School of Clinical Medicine, Southern Medical University, Guangzhou, China; ^5^School of Health in Social Science, University of Edinburgh, Edinburgh, United Kingdom; ^6^School of Nursing, Southern Medical University, Guangzhou, China; ^7^Guangdong Mental Health Center, Guangdong Provincial People's Hospital (Guangdong Academy of Medical Sciences), Guangzhou, China

**Keywords:** anxiety, breast cancer, depression, longitudinal, predictor, trajectory

## Abstract

**Background:**

The aim of the current study is to investigate the change in anxiety and depression amongst Chinese breast cancer patients and to identify causal associations between baseline variables and the trajectory of anxiety and depression within this identified group.

**Methods:**

This is a longitudinal prospective study. Three hundred women with breast cancer were recruited. Patient's depression and anxiety were repeatedly measured by PHQ-9 and GAD-7 at baseline, 6, 12, and 18 months after discharge. The SAS 9.4 PROC Traj procedure was used to examine the group-based trajectory of these recruited patients. Linear mixed models (LMM) were utilized to examine anxiety/depression changes over time, accounting for relevant baseline demographic and clinical factors.

**Results:**

About 26.3% of the participants reported none or very mild anxiety over time, 60.7% reported stable low-level anxiety, and the remaining 13.0% showed significantly decreasing trend in GAD total scores. Meanwhile, 10.7% of the participants reported none or very mild depressive symptoms over time, 66.0% reported stable PHQ total scores throughout the research period, and 23.3% were classified as the “high level-decreasing group”. Patients reported significantly higher anxiety and depression scores in the first three assessments. Participants with no or mild life stress along with a positive personality tended to report lower anxiety and depression scores over time.

**Conclusion:**

Most of the breast cancer patients reported stable low-level anxiety and depression 18 months after discharge. Early assessment of optimism and stress levels among cancer patients might help identify people at risk of experiencing long-term anxiety and depression.

## Introduction

Anxiety and depression have great influence on people's wellbeing, functioning, and productivity (1). A recent meta-analysis showed that the prevalence of anxiety amongst breast cancer patients could reach 41.9% (2). Another study reported that 45% of the women with breast cancer had severe levels of state anxiety at the time of cancer diagnosis (3). A prospective, multicenter cohort of 401 consecutive patients with newly diagnosed, advanced cancer showed that the incidence of anxiety was 36% during the COVID-19 pandemic (4). Those women who were married, non-religious, with higher monthly income, feelings of uncertainty and emotional stress were more likely to report anxiety than their counterparts (5). Anxiety significantly influences a patient's physiological and psychological functioning, treatment compliance and quality of life, and are significantly associated with cancer recurrence and all-cause mortality (6).

Depression is also common in people with physical illnesses. It is often under-estimated and under-treated amongst the cancer population (7). It has been reported that around 32.2% of the breast cancer patients experience depression after cancer diagnosis (8). Risk factors for depression among breast cancer patients include a previous history of major depressive disorder (MDD), loneliness, a higher number of comorbid conditions, financial difficulties, increased symptomatic burden, altered body image, alteration of femininity, sexuality as well as attractiveness (1, 9, 10). Additionally, breast cancer survivors with persistent breast pain are more likely to report higher levels of depressive symptoms (11, 12). Depression may influence the course of the disease and compliance, and is significantly associated with poor quality of life in cancer patients (7).

Comorbidity of anxiety and depression is common among breast cancer patients during treatment and survivorship (1). Sun et al., found that about 32.93% of the cancer patients experienced anxiety, and 38.55% reported depressive symptoms after diagnosis (1). Burgess et al., found that ~50% of the cancer survivors with early breast cancer diagnosis suffered from anxiety, depression, or both in the first year after diagnosis; the anxiety/depression rate dropped to 25% in the second year, and only 15% of the cancer survivors reported anxiety or depression in their fifth year after diagnosis (13). Boyes et al. investigated the prevalence and short-term trajectories of anxiety and depression in the first year after diagnosis, and found that 22 and 21% of the cancer patients reported anxiety 6 and 12 months after cancer diagnosis, and 13% reported depression at both timepoints (14).

Identifying and managing anxiety and depression amongst the cancer population is of great importance (6). Until now, there is an increasing body of research into cancer patient's anxiety and depression in China, however, information about the time course of anxiety and depression is limited because most previous studies have used cross-sectional research designs, with few of them concentrating on the patient's trajectory of anxiety and depression over long-term treatment and into survivorship. Among the few previously mentioned longitudinal studies; most span over a time period from soon after diagnosis to up to 1-year post-treatment. In this study, we aim to investigate the changes in anxiety and depression over a time frame of 18 months after discharge of this aforementioned group. Two research questions will be investigated: (1) what is the trajectory of anxiety and depressive symptoms in Chinese breast cancer patients? and (2) what are the predictors of change in anxiety and depression?

## Methods

### Study design and participants

This is a longitudinal prospective study consisting of 4 assessments at baseline (time of discharge), 6, 12, and 18 months after discharge. All breast cancer patients were consecutively recruited from January to December 2019 at Southern Medical University Nanfang Hospital and Guangdong Provincial People's Hospital. Participants were eligible if they were an adult; had a clear diagnosis of breast cancer; and were able to read, write and understand Chinese. Patients were excluded if they were under age; had obvious cognitive impairments; or receiving palliative treatment. This study's protocol was approved by Nanfang Hospital Ethics Committee (Ref no: NFEC-2018-038), and Guangdong Provincial People's Hospital Ethics Committee [Ref no: 2018295H(R1)]. Written informed consent was obtained from all participants on the day of recruitment. Participants were assured that their participation was voluntary, and that their information would be kept confidential. All participants were contacted by telephone or WeChat (a social communication application in China) by a research staff, to give their ratings again at follow-ups.

### Instruments

#### Personal information sheet

A specific set of questions were designed to collect information about patient's age, marital status, educational background, employment, monthly income, life stress level, physical comorbidities, family cancer history and personality tendency. Patient's life stress level was assessed using a self-designed question: what is your current stress level? Four responses were provided; namely: none, mild, moderate and high level. In addition, patient' personality tendency was measured by a single question derived from the Chinese version Personality Traits Questionnaire (15): what do you think is your dominant personality trait (Positive or Negative)?

#### Patient health questionnaire

The PHQ-9 is a self-report measure of 9 items used to assess depressive symptoms. Each item is rated between 0 and 3. Total PHQ-9 scores range from 0 to 27. A sum score of 5 or more suggests the presence of depressive symptoms (16). The Chinese PHQ-9 has good psychometric properties, with Cronbach's alpha of 0.89 (17).

#### Generalized anxiety disorder

The GAD-7 is a self-report measure of 7 items used to evaluate anxiety symptoms. Each item is rated from 0 and 3, with total scores ranging from 0 to 21. A sum GAD-7 score of 5 or more indicates the presence of anxiety symptoms (18). The Chinese GAD-7 has satisfactory psychometrics (19).

### Data analysis

All the statistical analyses were conducted with SAS version 9.4 (SAS Institute Inc., Cary. NC), and SPSS version 21.0 (SPSS Inc., Chicago, IL, USA). The SAS 9.4 PROC Traj procedure was used to investigate the group-based trajectory modeling and identify subgroups of patients who had similar trajectories on GAD and PHQ total scores over time. Censored normal (CNORM) model with cubic trajectory for each group was used for modeling the conditional distribution of GAD and PHQ scores. The model fit was assessed with Bayesian Information Criterion (BIC), and log Bayes factor [2loge(B10)≈2(ΔBIC)]. As recommended, the log Bayes factor of larger than 10 indicated a strong evidence of model fit (20).

To test the different distributions of baseline demographic and clinical variables among different trajectory groups, chi-square tests or Fisher's exact tests were performed. Furthermore, multinomial logistic regression analysis was conducted to explore the relationships between demographic and clinical characteristics and trajectory groups whilst adjusting for covariates. Additionally, the effects of baseline demographic and clinical variables on the changes of anxiety and depression (dependent variable) were examined *via* Generalized Linear Mixed Model by Restricted Maximum Likelihood (REML); with the fixed effects of time, age, marital status, education, employment, income, comorbidities, family history, current life stress, and personality, and random effects of subjects. The *post-hoc* comparisons of estimated marginal means of anxiety and depression scores over time were estimated with Bonferroni method. A two-side *P*-value < 0.05 was considered statistically significant.

## Results

### Patient characteristics

The mean age of participants was 44.94 years (SD = 9.79). At baseline, 144 (48.0%) of the participants reported depression, and 101 (33.7%) of the women reported anxiety. The mean score of PHQ decreased from 5.00 (SD = 4.26) at baseline (T1), to 4.21 (SD = 4.23) at 6-month follow-up (T2), 4.16 (SD = 3.33) at 12-month assessment (T3), and 3.24 (SD = 2.61) at 18-month follow-up endpoint (T4). Similarly, patients' anxiety level dropped from 3.50 (SD = 3.77) at baseline, to 3.26 (SD = 3.80) at T2, 3.15 (SD = 2.82) at T3, and 2.61 (SD = 2.77) at endpoint. The prevalence of anxiety and depression gradually decreased from 33.7 and 48.0% at baseline, 31.7 and 37.3% at T2, 22.3 and 26.9% at T3, to 13.9 and 14.6% at T4.

### Group-based trajectories and group comparisons

**Figure 1:**
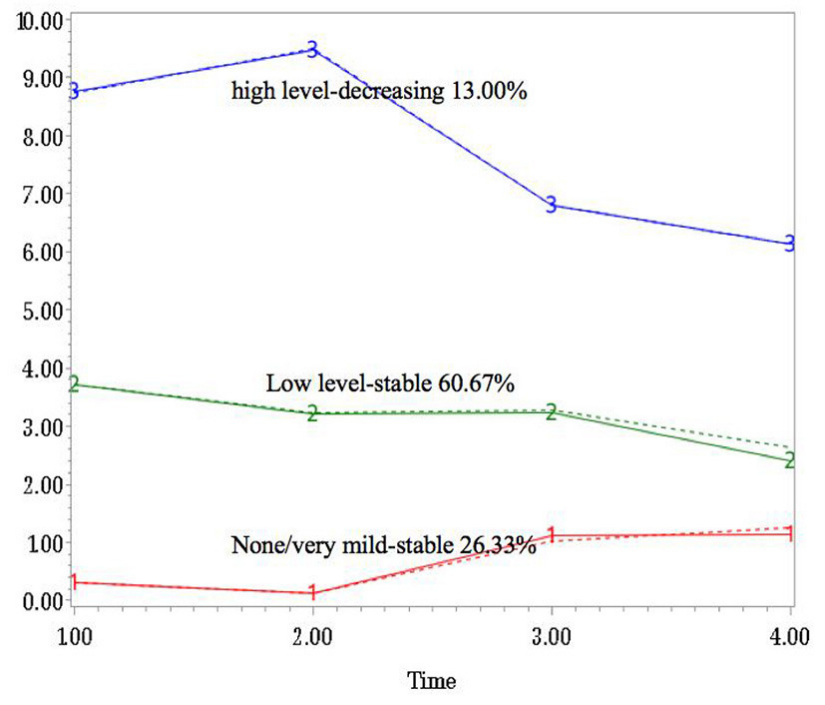
Group-based trajectories for the anxiety changes over time.

**Figure 2:**
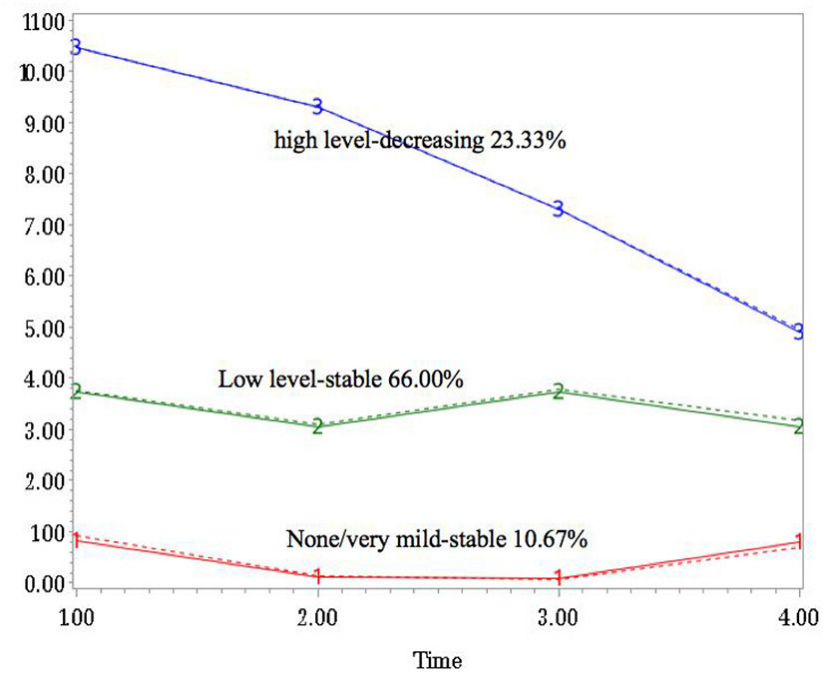
Group-based trajectories for the depression changes over time.

For both anxiety and depression, three-group trajectory models were estimated from the latent class growth modeling of SAS PROC Traj. The trajectories were labeled as: “none/very mild-stable group,” “low level-stable group,” and “high level-decreasing group” according to the significant difference of linear effect from 0, and the sign of coefficient (Figures 1, 2). For anxiety symptoms, 79 (26.3%) of the participants reported none or very mild anxiety over time, 182 (60.7%) reported stable low-level anxiety, and the remaining 39 (13.0%) showed apparent decreasing trend throughout the 18-month period. Similarly, 32 (10.7%) of the participants reported none or very mild depressive symptoms over time, 198 (66.0%) exhibited stable PHQ total scores throughout the research period, and 70 (23.3%) of the participants were in the “high level-decreasing trajectory group”.

Chi-square tests showed that participant's monthly income (X^2^ = 13.156, *P* = 0.041), life stress (X^2^ = 15.300, *P* = 0.018) and personality (X^2^ = 28.478, *P* < 0.001) were significantly differently distributed among the three anxiety groups. Also, participant's physical comorbidities (X^2^ = 9.989, *P* = 0.007), life stress (X^2^ = 18.865, *P* = 0.004) and personality (X^2^ = 16.511, *P* < 0.001) were significantly differently distributed among three depression groups. Other sociodemographic and clinical variables had no significant associations with anxiety/depression trajectory groups (Table 1).

**Table 1:** Demographic and clinical characteristics of participants among different trajectory groups (*N* = 300).

**Variable**	**Total**	**Anxiety**					**Depression**				
	***N* (%)**	**Group 1**	**Group 2**	**Group 3**	**X^2^/Z**	***P***	**Group 1**	**Group 2**	**Group 3**	**X^2^/Z**	***P***
**Age**											
Below 39 years	93 (31.0)	22 (27.8)	58 (31.9)	13 (33.3)	1.942	0.746	10 (31.3)	60 (30.3)	23 (32.9)	5.530	0.237
40–59 years	176 (58.7)	51 (64.6)	104 (57.1)	21 (53.8)			20 (62.5)	121 (61.1)	35 (50.0)		
60 and above	31 (10.3)	6 (7.6)	20 (11.0)	5 (12.8)			2 (6.3)	17 (8.6)	12 (17.1)		
**Marital status**											
Single	19 (6.3)	3 (3.8)	15 (8.2)	1 (2.6)	9.033	0.172	1 (3.1)	15 (7.6)	3 (4.3)	6.716	0.348
Married	259 (86.3)	71 (89.9)	154 (84.6)	34 (87.2)			27 (84.4)	171(86.4)	61 (87.1)		
Divorced	17 (5.7)	2 (2.5)	12 (6.6)	3 (7.7)			2 (6.3)	11 (5.6)	4 (5.7)		
Widowed	5 (1.7)	3 (3.8)	1 (0.5)	1 (2.6)			2 (6.3)	1 (0.5)	2 (2.9)		
**Education**											
Primary and middle school	124 (41.3)	24 (30.4)	80 (44.0)	20 (51.3)	6.096	0.192	9 (28.1)	80 (40.4)	35 (50.0)	5.812	0.214
High school	108 (36.0)	33 (41.8)	63 (34.6)	12 (30.8)			12 (37.5)	75 (37.9)	21 (30.0)		
Undergraduate and above	68 (22.7)	22 (27.8)	39 (21.4)	7 (17.9)			11 (34.4)	43 (21.7)	14 (20.0)		
**Employment**											
Full time	146 (48.7)	47 (59.5)	82 (45.1)	17 (43.6)	9.147	0.165	19 (59.4)	98 (49.5)	29 (41.4)	3.740	0.712
Part time	26 (8.7)	2 (2.5)	20 (11.0)	4 (10.3)			2 (6.3)	18 (9.1)	6 (8.6)		
Unemployment	80 (26.6)	17 (21.5)	53 (29.1)	10 (25.6)			8 (25.0)	50 (25.3)	22 (31.4)		
Retired	48 (16.0)	13 (16.5)	27 (14.8)	8 (20.5)			3 (9.4)	32 (16.2)	13 (18.6)		
**Monthly income**											
< 3,000 RMB	116 (38.7)	23 (29.1)	79 (43.4)	14 (35.9)	13.156	**0.041**	9 (28.1)	73 (36.9)	34 (48.6)	9.171	0.164
3,000–5,000 RMB	79 (26.3)	19 (24.1)	47 (25.8)	13 (33.3)			9 (28.1)	51 (25.8)	19 (27.1)		
5,000–10,000 RMB	68 (22.7)	27 (34.2)	31 (17.0)	10 (25.6)			10 (31.3)	44 (22.2)	14 (20.0)		
More than 10,000 RMB	37 (12.3)	10 (12.7)	25 (13.7)	2 (5.1)			4 (12.5)	30 (15.2)	3 (4.3)		
**Chemotherapy**											
Yes	260 (86.7)	71 (89.9)	155 (85.2)	34 (87.2)	1.067	0.587	30 (93.8)	167 (84.3)	63 (90.0)	2.987	0.225
No	40(13.3)	8 (10.1)	27 (14.8)	5 (12.8)			2 (6.3)	31 (15.7)	7 (10.0)		
**Physical comorbidities**											
Yes	230 (76.7)	67 (84.8)	137 (75.3)	26 (76.7)	5.306	0.070	27 (84.4)	159(80.3)	44 (62.9)	9.989	**0.007**
No	70 (23.3)	12 (15.2)	45 (24.7)	13 (33.3)			5 (15.6)	39 (19.7)	26 (37.1)		
**Family cancer history**											
Yes	57 (19.0)	11 (13.9)	35 (19.2)	11 (28.2)	3.476	0.176	5 (15.6)	37 (18.7)	15 (21.4)	0.581	0.772
No	243 (81.0)	68 (86.1)	147 (80.8)	28 (71.8)			27 (84.4)	161(81.3)	55 (78.6)		
**Life stress**											
None	78 (26.0)	28 (35.4)	42 (23.1)	8 (20.5)	15.300	**0.018**	13 (40.6)	54 (27.3)	11 (15.7)	18.865	**0.004**
Mild	115 (38.3)	36 (45.6)	67 (36.8)	12 (30.8)			11 (34.4)	84 (42.4)	20 (28.6)		
Moderate	69 (23.0)	11 (13.9)	45 (24.7)	13 (33.3)			5 (15.6)	39 (19.7)	25 (35.7)		
High	38 (12.7)	4 (5.1)	28 (15.4)	6 (15.4)			3 (9.4)	21 (10.6)	14 (20.0)		
**Personality**											
Positive	251 (83.7)	77 (97.5)	151 (83.0)	23 (59.0)	28.478	**< 0.001**	32(100.0)	170(85.9)	49 (70.0)	16.511	**< 0.001**
Negative	49 (16.3)	2 (2.5)	31 (17.0)	16 (41.0)			0 (0.0)	28 (14.1)	21 (30.0)		

1 US Dollar ≈ 6.3 RMB. Group 1: none/mild-stable; Group 2: low level-stable; Group 3: High level-decreasing. Bold values means statistically significant.

Multinomial logistic regression revealed that, compared with the “none/very mild-stable anxiety group”, baseline life stress and negative personality were positively associated with low level-stable anxiety group (OR = 1.69, 95% CI = 1.18–2.43, *P* = 0.004, and OR = 7.24, 95% CI = 1.59–32.78, *P* = 0.010, respectively) and high-level decreasing anxiety group (OR = 1.85, 95% CI = 1.12–3.07, *P* = 0.017, and OR = 24.91, 95% CI = 4.94–125.61, *P* < 0.001, respectively) after adjusting for covariates. Similarly, compared to the high-level decreasing depression group, patients in the low level depression group were less likely to report severe life stress (OR = 0.654, 95% CI = 0.467–0.916, *P* = 0.013) and negative personality (OR = 0.448, 95% CI = 0.219–0.915, *P* = 0.028). Patients in none/very mild depression group were also less likely to suffer from high life stress compared to the high-level group (OR = 0.469, 95% CI = 0.282–0.872, *P* = 0.015).

### Associations of baseline variables on the trajectory changes in linear mixed models

The linear mixed models showed that time (*F* = 7.728, *P* < 0.001; and *F* = 20.098, *P* < 0.001), life stress level (*F* = 6.588, *P* < 0.001; and *F* = 5.731, P=0.001), and personality (*F* = 23.305, *P* < 0.001; and *F* = 21.883, *P* < 0.001) significantly affected both anxiety and depression. Specifically, Bonferroni-corrected post-hoc tests revealed that patients reported significantly higher anxiety score at baseline (T1, estimate = 0.918, 95% CI: 0.452–1.384, *P* < 0.001), the 2nd (T2, estimate = 0.681, 95% CI: 0.261–1.101, *P* = 0.002), and 3rd measurements (T3, estimate = 0.571, 95% CI: 0.304–0.839, *P* < 0.001), than at the endpoint assessment (T4). Compared with those with high levels of life stress, patients who had no stress [estimate = −1.789, 95% CI: −2.824 – (−0.754), *P* = 0.001] or mild stress [estimate = −1.802, 95% CI: −2.748 – (−0.856), *P* < 0.001] would perform at a significantly lower anxiety level. Also, patients who had a positive personality would exhibit significantly less anxiety symptoms than their counterparts [estimate = −1.821, 95% CI: −2.563 – (−1.078), *P* < 0.001] (Table 2). Similar results were found in patient's depression trajectory. Patients reported significantly higher depression scores at the time of the first three assessments than at the fourth measurements (*P* all < 0.001). Participants with no (*P* = 0.001) or mild life stress (*P* = 0.003) would also then report lower depression scores than those with high stress level. In addition, patients who had positive personality tended to report lower depression scores compared to their counterparts (*P* < 0.001) (Table 3).

**Table 2:** Associations between baseline variables on the changes of anxiety in linear mixed models.

**Parameters**	**Comparisons**	**Estimates**	**P value**	**95% CIs**	
				**Lower**	**Upper**
Age (years)	Below 39 years	0.986	0.872	−1.108	1.305
	40–59 years	−0.501	0.353	−1.563	0.560
	60 and above	Ref	–	–	–
Marital status	Single	1.571	0.201	−0.840	3.983
	Married	1.928	0.075	−0.195	4.052
	Divorced	2.102	0.084	−0.284	4.489
	Widowed	Ref	–	–	–
Education	Primary and middle school	0.173	0.664	−0.611	0.957
	High school	0.630	0.087	−0.092	1.353
	Undergraduate and above	Ref	–	–	–
Employment	Full time	−0.515	0.292	−1.476	0.446
	Part time	0.420	0.501	−0.807	1.647
	Unemployment	−0.770	0.155	−1.832	0.293
	Retired	Ref	–	–	–
Monthly income	< 3,000 RMB	0.448	0.433	−0.676	1.572
	3,000–5,000 RMB	0.215	0.660	−0.746	1.177
	5,000–10,000 RMB	0.362	0.442	−0.564	1.288
	More than 10,000 RMB	Ref	–	–	–
Chemotherapy	Yes	0.125	0.763	−0.689	0.939
	No	Ref	–	–	–
Physical comorbidities	Yes	−0.424	0.204	−1.079	0.232
	No	Ref	–	–	–
Family cancer history	Yes	0.249	0.485	−0.453	0.951
	No	Ref	–	–	–
Life stress	None	−1.789	**0.001**	−2.824	−0.754
	Mild	−1.802	**< 0.001**	−2.748	−0.856
	Moderate	−0.648	0.180	−1.598	0.301
	High	Ref	–	–	–
Personality	Positive	−1.821	**< 0.001**	−2.563	−1.078
	Negative	Ref	–	–	–
Timepoint	1	0.918	**< 0.001**	0.452	1.384
	2	0.681	**0.002**	0.261	1.101
	3	0.571	**< 0.001**	0.304	0.839
	4	Ref	–	–	–

Bold: statistically significant; 1 US dollar ≈ 6.3 RMB.

**Table 3:** Associations between baseline variables on the changes of depression in linear mixed models.

**Parameters**	**Comparisons**	**Estimates**	**P value**	**95% CIs**	
				**Lower**	**Upper**
Age (years)	Below 39 years	0.831	0.190	−0.415	2.077
	40–59 years	0.387	0.487	−0.710	1.485
	60 and above	Ref	–	–	–
Marital status	Single	0.650	0.604	−1.815	3.115
	Married	0.838	0.448	−1.332	3.008
	Divorced	1.234	0.321	−1.210	3.677
	Widowed	Ref	–	–	–
Education	Primary and middle school	0.008	0.984	−0.800	0.816
	High school	0.196	0.602	−0.543	0.935
	Undergraduate and above	Ref	–	–	–
Employment	Full time	−0.620	0.221	−1.615	0.375
	Part time	−0.271	0.674	−1.540	0.998
	Unemployment	−0.756	0.176	−1.852	0.340
	Retired	Ref	–	–	–
Monthly income	< 3,000 RMB	0.749	0.206	−0.413	1.912
	3,000–5,000 RMB	0.457	0.362	−0.529	1.444
	5,000–10,000 RMB	0.481	0.319	−0.467	1.429
	More than 10,000 RMB	Ref	–	–	–
Chemotherapy	Yes	0.066	0.877	−0.769	0.901
	No	Ref	–	–	–
Physical comorbidities	Yes	−0.571	0.096	−1.243	0.101
	No	Ref	–	–	–
Family cancer history	Yes	0.479	0.192	−0.242	1.200
	No	Ref	–	–	–
Life stress	None	−1.787	**0.001**	−2.847	−0.727
	Mild	−1.457	**0.003**	−2.430	−0.484
	Moderate	−0.404	0.415	−1.379	0.570
	High	Ref	–	–	–
Personality	Positive	−1.820	**< 0.001**	−2.585	−1.054
	Negative	Ref	–	–	–
Timepoint	1	1.747	**< 0.001**	1.264	2.231
	2	0.964	**< 0.001**	0.512	1.416
	3	0.922	**< 0.001**	0.579	1.264
	4	Ref	–	–	–

Bold: statistically significant; 1 US dollar ≈ 6.3 RMB.

## Discussion

The prevalence of anxiety and depression in women with breast cancer was found to be 33.7 and 48.0% at discharge, and 13.9 and 14.6% at 18-month assessment. Our findings were in line with Burgess's study which reported that the prevalence of anxiety and depression amongst women with breast cancer declined from 48 to 15% from the first year of diagnosis to the fifth year after diagnosis (13). Cancer diagnosis and its treatment could be conceptualized as traumatic events and lead to long-term psychological disturbances (21). Previous studies reported that about 30% of patients suffer from anxiety or depression for almost 1 year after diagnosis (22–24), and the prevalence estimates of persistent depression range from 12 to 25% among adult cancer patients (13, 25). Another study found that even 20 years after diagnosis, 5% of breast cancer survivors still suffer from psychological distress (26).

In this study, 60.7 and 66.0% of the women reported stable low-level anxiety and depression throughout the 18-month period after treatment. Meanwhile, 13.0 and 23.3% showed a decreasing trend in anxiety and depression. Our research findings are partially consistent with past evidence indicating that the levels of cancer patient's anxiety and depression usually gradually decrease or remain stable. For example, Saboonchi et al. investigated the trajectories of anxiety in 725 breast cancer survivors over a 2-year follow-up, and successfully identified four different trajectories (i.e., High Stable, High Decrease, Mild Decrease and Low Decrease) (27). Ng et al. also found that there is significant reduction in patient's anxiety level at 6 and 12 months as compared to baseline (28), while Kristin et al. reported that the anxiety level in 236 post-surgery breast cancer patients remained unchanged after 1 year (29). As for depression, Avis et al., evaluated the trajectories of depressive symptoms following breast cancer diagnosis and found that most breast cancer patients could be classified as having very low (3.8%) or low (47.3%) depressive symptoms at all of the timepoints, while 11.3% of the patients had initially high scores that declined over 24 months after diagnosis (30). Another longitudinal study also found that there was significant reduction in depressive symptoms over the 14-month follow-up period (31). However, in a 1-year prospective study, depression was found to be relatively low and did not change significantly at both 6 and 12 months timepoint (28).

In line with previous publications, in this study, participants with none or mild life stress tended to report lower anxiety and depression scores over time. According to previous evidence, stressful life events predispose cancer patients in developing anxiety, depression and other emotional disturbances (13, 32, 33). One possible reason is that stressful events waste patients' psychological energy and exhaust their coping resources that would otherwise be used to deal with their psychological problems (34). The relationship between stress and internalizing psychopathologies has been strongly established (35). Past studies indicated that stressful life events predict depressive episodes and are significantly associated with anxiety amongst different populations (35). Compared to their counterparts, depressed or anxious individuals tend to exhibit helplessness behaviors toward stressors; therefore, interventions such as stress control psycho-educational program might help in reducing a patient's psychological distress (36).

Another possible reason why some patients tend to have better adjustment than others is their personality trait. Researchers have suggested that personality is one of the most significant factors when coping with life-threatening illness, and optimism may be a key factor in understanding why some people experience long-term anxiety and depression (37). There are a number of studies indicating that the level of optimism is negatively correlated with anxiety and depression (38). Compared to pessimists, optimists tend to report better psychological resilience (e.g., better problem-solving coping), wellbeing, and health-related quality of life (21). A recent similar study indicated that lower level of optimism at the time of breast cancer diagnosis significantly predicted the development of both anxiety and depression 5 years after diagnosis. Prince et al. examined the association between neuroticism/extraversion and first-onset anxiety and depressive disorders and found that high neuroticism is either a significant risk factor, or a marker of risk, for anxiety and depressive disorders (39). Results also suggested that individuals who reported lower levels of trait positive emotionality were more likely to have a chronic course of depression (40). One possible reason why optimism influences long-term psychological distress is that it affects the way cancer patients cope with the diagnosis, treatment, progression, and other serious events (21). Therefore, early assessment of optimism levels among cancer patients might help to identify women at risk of experiencing long-term anxiety and depression (21).

Several limitations of this study merit consideration. First, it is important to recognize that these results may not apply to all women diagnosed with cancer as only women with breast cancer were included in the current study. Second, several covariates (e.g., job loss, and the experience of psychotherapy) were not investigated, findings should not be generalized without considering the possible effects of these factors. Third, single-item self-report personality measurement may not adequately examine underlying personality features of individuals. Further investigations of personality are warranted as a single item provides weak psychometric information.

## Conclusions

Most of the breast cancer patients reported stable low-level anxiety and depression 18 months after discharge. Life stress and personality traits helps predict course trajectories for anxiety and depression. Early assessment of optimism and stress levels among cancer patients might help to identify people at risk of experiencing long-term anxiety and depression.

## Data availability statement

The raw data supporting the conclusions of this article will be made available by the authors, without undue reservation.

## Ethics statement

The studies involving human participants were reviewed and approved by Nanfang Hospital Research Ethics Committee (Ref no: NFEC-2018-038), and Guangdong Provincial People's Hospital Research Ethics Committee [Ref no: 2018295H(R1)]. The patients/participants provided their written informed consent to participate in this study.

## Author contributions

WL, YC, and YY: study design. WL, QZ, YX, HS, YW, WX, and YT: data collection, analysis, and interpretation. WL, QZ, and YX: drafting of the manuscript. SG, YC, and YY: critical revision of the manuscript. All authors: approval of the final version for publication.

## Funding

This research was supported in part by the start-up funds of Guangdong Provincial People's Hospital (KY0120211134), the construction project of Team Construction in Guangdong Province Teaching Quality and Teaching Reform Project (Higher Education in Guangzhou [2020] 19), College Innovation and Entrepreneurship (Employment) Education Project in Guangzhou (Higher Education in Guangzhou [2019] 15), and Guangdong Province Degree and Postgraduate Education Reform Research Project (2021JGXM026).

## Conflict of interest

The authors declare that the research was conducted in the absence of any commercial or financial relationships that could be construed as a potential conflict of interest.

## Publisher's note

All claims expressed in this article are solely those of the authors and do not necessarily represent those of their affiliated organizations, or those of the publisher, the editors and the reviewers. Any product that may be evaluated in this article, or claim that may be made by its manufacturer, is not guaranteed or endorsed by the publisher.
